# Urinary Calcium Is Associated with Serum Sclerostin among Stone Formers

**DOI:** 10.3390/jcm12155027

**Published:** 2023-07-31

**Authors:** Fernanda Guedes Rodrigues, Milene Subtil Ormanji, Igor Gouveia Pietrobom, Ana Cristina Carvalho de Matos, Martin H. De Borst, Ita Pfeferman Heilberg

**Affiliations:** 1 Nutrition Post Graduation Program, Universidade Federal de São Paulo, São Paulo 04023-062, Brazil; ita.heilberg@gmail.com; 2 Department of Nephrology, University Medical Center Groningen, University of Groningen, 9713 GZ Groningen, The Netherlands; m.h.de.borst@umcg.nl; 3 Division of Nephrology, Universidade Federal de São Paulo, São Paulo 04023-062, Brazil; milene.ormanji@gmail.com (M.S.O.); igorpietrobom@gmail.com (I.G.P.); anacrismatos21@gmail.com (A.C.C.d.M.)

**Keywords:** kidney stones, urinary calcium, serum sclerostin, bone mineral density

## Abstract

Background: Sclerostin plays an important role in bone metabolism and adipose tissue. Animal studies suggest that sclerostin influences urinary calcium (UCa), but this relationship has not been evaluated in stone formers (SFs). We aimed to investigate the association of UCa with serum sclerostin, bone mineral density (BMD), and body composition among SFs. Methods: Clinical and laboratorial data were retrieved from medical records. Determinants of UCa were studied using linear regression. Results: A total of 107 SFs (35.8 ± 9.3 years, 54% male) with eGFR 99.8 ± 14.5 mL/min/1.73 were studied. Subjects were split by sex and grouped into tertiles of UCa levels. Men in the highest UCa tertile had higher body mass index (BMI) and serum sclerostin, lower lean mass, and a trend towards higher fat mass. Women in the highest tertile had higher BMI and a trend towards higher serum sclerostin. Hypertension and metabolic syndrome, but not lower BMD, were more prevalent in the highest UCa tertile for both sexes. Sclerostin was positively correlated with fat mass and inversely correlated with lean mass among men, but not among women. BMD corrected for BMI at lumbar spine was inversely associated with UCa in a univariate analysis, but only serum sclerostin, hypertension, and NaCl intake were independent determinants of UCa in the multivariate model. Conclusion: The present findings disclose that in addition to hypertension and salt intake, serum sclerostin is associated with urinary calcium in stone formers, suggesting that in addition to the hormones traditionally thought to alter calcium reabsorption in the kidney, sclerostin may play a significant additional role, warranting further investigation.

## 1. Background

Hypercalciuria is the most common metabolic abnormality found in nearly 50% of calcium stone formers and characterized as a systemic abnormality of calcium homeostasis in which a dysregulation of calcium transport takes place in the kidneys, intestine, and bones, under influence of genetics, diet, and hormones, such as parathyroid hormone (PTH), fibroblast growth factor-23 (FGF-23), and the active form of vitamin D_3_, 1,25-dihydroxyvitamin-D_3_ (1,25(OH)_2_D_3_) [1,2,3].

Increased body mass index (BMI) has been associated with alterations in urinary composition, including enhanced urinary calcium excretion [4]. A previous study of our group in stone-forming patients has disclosed a positive correlation of urinary calcium excretion not only with BMI, but also with waist circumference [5], suggesting an effect of body composition on calciuria.

Stone formers (SFs) exhibit reduced bone mineral density (BMD) [6,7], and previous histomorphometric studies have evidenced low bone formation and increased bone resorption [8,9]. Although bone demineralization occurs mostly in SFs with idiopathic hypercalciuria [8,9,10], it may be detected in normocalciuric SFs as well [11,12].

Sclerostin, a glycoprotein expressed by osteocytes as an inhibitor of the Wnt-β catenin pathway, is a negative regulator of bone formation [13], and its circulating levels exhibit a positive association with BMI and fat mass in healthy subjects [14,15]. It follows that *Sost* gene (which encodes sclerostin) knockout (KO) mice have reduced fat mass and adipocyte size [16] as well as increased BMD due to greater bone formation [17]. Interestingly, another experimental study showed that mice with sclerostin gene deletion exhibited a reduction in urinary calcium levels [18]. To date, little is known about the effects of circulating sclerostin upon urinary calcium among SFs. Therefore, we aimed to characterize a possible relationship of urinary calcium levels with circulating sclerostin, BMD, and body composition in this population.

## 2. Methods

### 2.1. Study Population

Adult stone-forming patients, referred to the Nephrolithiasis Outpatient Clinic of the Universidade Federal de São Paulo (UNIFESP) because of an established diagnosis of renal stone, were enrolled in the present retrospective study with cross-sectional analysis. A diagnosis of nephrolithiasis was made based on the presence of renal colic with hematuria, spontaneous calculi voiding, and/or surgical/endoscopic removal of the stones and/or radiographic evidence of stone(s). 

The present study is based on available data retrieved from their medical records regarding clinical characteristics, nutritional and biochemistry data, as well as bone mineral density (BMD) and body composition determined by dual energy X-ray absorptiometry (DXA). Patients had DXA and biobank available due to participation in a previous study. Metabolic syndrome was diagnosed according to the American Heart Association criteria. Serum levels of sclerostin, 1,25 dihydroxyvitamin D_3_ (1,25(OH)_2_D_3_), fibroblast growth factor 23 (FGF-23), and klotho were presently measured in their stored blood samples whenever available. 

Exclusion criteria were men over 60 years old, postmenopausal women, estimated glomerular filtration rate (eGFR) by the Chronic Kidney Disease Epidemiology Collaboration (CKD-EPI) equation < 90 mL/min/1.73 m^2^, diabetes mellitus, renal tubular acidosis, hyperparathyroidism, or past use of thiazides, corticosteroids, and anticonvulsants. 

Written consent was obtained from each patient, and the study protocol was approved by the local Medical Ethics and Research Committee of UNIFESP (number 4.869.310) in accordance with the Helsinki Declaration of 1975. 

### 2.2. Bone Mineral Density and Body Composition 

Bone densitometry was assessed by DXA (QDR 4500, Hologic Inc., Waltham, MA, USA) with measurements of BMD at lumbar spine, femoral neck, and total femur. Fat mass and lean mass were also determined by DXA, and the percentage of both was calculated by total body weight. Anthropometric parameters (weight and standing height) were used to calculate body mass index (BMI). To correct for the influence of the weight load on bone, the lumbar spine, femoral neck, and total femur were also expressed as a ratio corrected for BMI (BMD/BMI) [19].

### 2.3. Nutritional Data 

Micronutrients intake, such as calcium, phosphorus, and potassium, was obtained by a 3-day food-intake record under unrestricted diet, and their daily consumption was adjusted for the total energy intake and calculated using the Dietpro—version 6.0 software (USDA nutrients data). Sodium chloride (NaCl) intake was estimated from 24 h urine sodium excretion and the protein intake from 24 h urea excretion using the protein equivalent of nitrogen appearance (PNA) formula. PNA = (urinary urea nitrogen + [0.031 × weight (kg)]) × 6.25, where urinary urea nitrogen is (urinary urea (mg/L/24 h)/2.14 × urinary volume in 24 h (L)).

### 2.4. Biochemical Parameters

The serum parameters analyzed in stored blood samples were sclerostin, 1,25(OH)_2_D_3_, FGF-23, and klotho. The 24-hr urinary biochemistry (calcium, oxalate, phosphate, sodium, potassium and urea) and the remaining serum biochemical and hormonal parameters (creatinine, 25(OH)D3, PTH, and bone alkaline phosphatase (BAP) were obtained from their medical records. Serum 25(OH)D_3_, 1,25(OH)_2_D_3_, and PTH were determined by a chemiluminescence assay (Architect i200 SR, Abbott). Serum FGF-23, Klotho, sclerostin, and BAP were quantified using ELISA kits (Immutopics Inc., USA; IBL, USA; Teco Medical, Sissach, Switzerland; Quidel, San Diego, CA, USA; respectively). Urinary oxalate was determined via an enzymatic method (Trinity Biotech, Bray, Ireland). Urinary and serum creatinine was determined according to the modified Jaffe’s reaction. Urinary calcium and phosphate were determined by a colorimetric method and sodium and potassium by an ion-selective electrode. All biochemical parameters were measured in a Beckman Clinical Chemistry Analyzer (AU480- Beckman Coulter, Brea, CA, USA). The eGFR was calculated using the 2022 Chronic Kidney Disease Epidemiology Collaboration (CKD-EPI) equation. 

### 2.5. Statistical Analysis

Statistical analyses were performed using IBM SPSS version 23.0 (SPSS Inc., Chicago, IL, USA). In all analyses, *p* < 0.05 was considered significant. Variable distribution was evaluated via the Kolmogorov–Smirnov test. Categorical variables are presented as n (%), normally distributed variables as mean ± standard deviation (SD), and non-normally distributed variables as median (interquartile range). Comparison of categorical variables was performed using a Chi-square test. Differences in tertiles were tested through an analysis of variance (ANOVA) with the Bonferroni post hoc tests for normally distributed variables and the Kruskal–Wallis test for non-normal distribution. Possible determinants of urinary calcium were studied using univariate linear regression. Since we aimed to explore the potential relevance of clinical and nutritional factors as potential determinants, we tested all variables of known clinical importance for urinary calcium (i.e., body composition, dietary sodium and protein, presence of hypertension and metabolic syndrome, serum PTH and BAP, FGF-23, sclerostin, 1,25(OH)_2_D_3_, and 25(OH)D_3_) in univariate regression analysis. Subsequently, all variables with a *p* < 0.10 were included in a multivariate linear regression model to identify the independent determinants of urinary calcium. Predictors were tested for collinearity using variance inflation factor analysis. Residuals were checked for normality and were natural log-transformed when appropriate. Co-adjustment for energy was performed for all nutrients using the residual method.

## 3. Results 

Clinical characteristics and nutritional data of the participants included in the analyses are shown in Table 1. The subjects were divided by sex and clustered into three groups according to tertiles (T) of urinary calcium. Rationale for separating patients according to sex was the well-established differences in body composition and BMD for men and women. A total of 107 (one hundred and seven) SFs were included in the study with a mean age of 35.8 ± 9.3 years. There was no statistical difference regarding age and duration of disease across tertiles. Among men, means of BMI were higher in T3 when compared to T1 and T2, while the percentage of lean mass was higher in the former. Among women, T3 presented higher BMI when compared to T2. Among both sexes, there was a higher prevalence of hypertension and metabolic syndrome in T3 than T1 and T2. With respect to nutritional data, there were no differences between groups regarding intakes of protein, NaCl, calcium, phosphorus, and potassium among men. However, among women, T3 presented the higher intake of NaCl.

BMD and biochemistry parameters are shown in Table 2. There were no statistical differences between the groups regarding BMD in the three sites (lumbar spine, femoral neck, and total femur). Phosphaturia was higher in T3 when compared to T1 and T2 among men and women. There was no statistical difference in both sexes regarding urinary oxalate, potassium, and eGFR.

Regarding serum parameters, circulating sclerostin levels were higher among men in T3 compared to T1 and T2 (30.9 pmol/L [25.4–37.1] vs. 21.4 pmol/L [18.3–26.7] and 28.8 pmol/L [19.0–40.9], *p* < 0.001, respectively). Among women, T3 presented a trend for higher serum sclerostin versus T1 (25.9 [17.5–29.4] vs. 19.8 [11.0–23.9], *p* = 0.06). There were no statistical differences between groups in both sexes regarding 25(OH)D_3_, 1,25(OH)_2_D_3_, PTH, BAP, FGF-23, and klotho serum levels.

To correct for the influence of the weight load on bone, the lumbar spine BMD, femoral neck BMD, and total femur BMD were corrected for BMI by calculating their ratios, the mean values of which were 0.037 ± 0.006, 0.030 ± 0.006, and 0.036 ± 0.006, respectively (data not shown in tables).

We subsequently performed linear regression to investigate possible clinical, laboratory, and dietary factors as determinants of urinary calcium. Associations were explored for already established factors that influence urinary calcium. The linear regression analysis results are presented in Table 3. Upon univariate analysis, we observed significant positive associations between urinary calcium and age (st. β 0.26, *p* < 0.01), presence of hypertension (st. β 0.33, *p* < 0.01), NaCl intake (st. β 0.31, *p* < 0.01), serum sclerostin (st. β 0.31, *p* < 0.01), and a negative association with the lumbar spine BMD/BMI ratio (st. β −0.24, *p* = 0.02). In multivariate linear regression analyses, the presence of hypertension, NaCl intake, and serum sclerostin remained strongly associated with urinary calcium (st. β 0.30, *p* < 0.01; st. β 0.31, *p* = 0.02; st. β 0.26, *p* = 0.01, respectively). 

**Figure 1:**
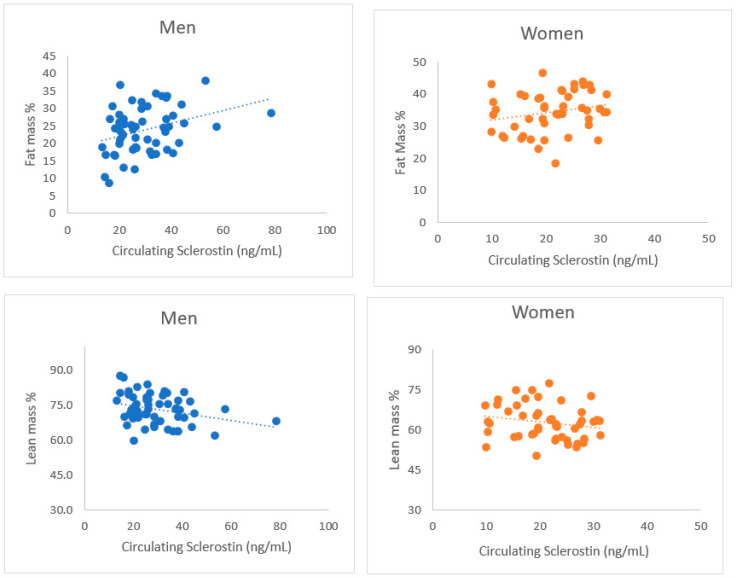
Association scatterplots of serum sclerostin levels with fat mass % (Men: β 0.38, *p* = 0.004; Women: β 0.22, *p* = 0.14) and lean mass % (Men: β –0.32, *p* = 0.01; Women: β –0.20, *p* = 0.17).

In the current study, circulating sclerostin was associated with body composition among male SFs. As shown in Figure 1, serum sclerostin levels were positively associated with fat mass % among men (β 0.38, *p* = 0.004), but not in women (β 0.22, *p* = 0.14). Further, it was negatively associated with body lean mass % among men (β –0.32, *p* = 0.01), but not among women (β –0.20, *p* = 0.17).

## 4. Discussion

In the present study, both male and female stone formers with higher urinary calcium excretion exhibited higher prevalence of hypertension, metabolic syndrome, and higher BMI. Among male SFs, a significantly higher fat mass % and lower lean mass % were evidenced in parallel with increased calciuria and circulating levels of sclerostin. A trend for higher serum sclerostin was also disclosed in female SFs with more elevated urinary calcium levels. Notably, serum sclerostin levels, hypertension, and salt intake were shown to be independent determinants of urinary calcium. 

The present association of BMI with urinary calcium agrees well with previous findings of a larger cohort from our group showing correlations with waist circumference as well [5] and with data reported by Shavit et al. [20], which emphasized that obese and overweight SFs patients have higher urinary calcium. 

There was a higher prevalence of hypertensive patients in the highest tertile of calciuria in both sexes in the current study, with hypertension ending up as an independent determinant of urinary calcium in a multivariate linear regression. Hypertension has already been associated with abnormalities in calcium metabolism and renal tubular calcium handling resulting in an increased urinary calcium [21]. 

The current independent and positive association of NaCl intake with urinary calcium levels is in line with previous studies [22]. This effect is attributed to the complex interplay between sodium and calcium transport mechanisms in the kidneys. A high sodium diet suppresses both proximal sodium and calcium reabsorption increasing the delivery of calcium to the distal nephron, exceeding its transport capacity, resulting in hypercalciuria [23]. In addition, prolonged exposure to high dietary NaCl may have implications for bone loss in stone formers as well. [24]. 

In the present series, circulating sclerostin levels were higher among patients in the highest tertiles of was the urinary calcium (tertiles 2 and 3).. A trend for higher level of sclerostin among female SFs was found in the T3 of urinary calcium (*p* = 0.06). In addition, the multivariate regression analysis disclosed circulating sclerostin as a strong determinant of urinary calcium, even when adjusting for sex (β 0.30, *p* < 0.01). The way by which sclerostin may influence calcium reabsorption in the distal tubule has been investigated in an elegant experimental study by Ryan et al. [17] who showed that *Sost* KO mice had lower urinary calcium, increased serum levels of 1,25(OH)_2_D_3_ and phosphorus, and decreased FGF-23, which may have contributed to the regulation of mineral accretion, resulting in increased BMD observed in these animals. Sclerostin indirect effects on calciuria could be mediated by the inhibition of CYP27B1 expression leading to the lower synthesis of 1,25(OH)_2_D_3_ or by the stimulation of the release of FGF-23 in bone, which in turn would inhibit the renal production of 1,25(OH)_2_D_3_ [18]. The FGF-23 concentration was indeed diminished in *Sost* KO mice along with an increase in serum phosphate concentrations [17]. Since 1,25(OH)_2_D_3_ stimulates Ca reabsorption at distal tubule, the inhibitory effects of its synthesis are expected to favor Ca excretion. Interestingly, PTH appears to inhibit sclerostin concentrations, as evidenced by its effects decreasing *Sost* expression in primary cultures of murine calvaria cells [25] as a hormonal control of osteoblastogenesis. However, we have not observed significant differences among 1,25(OH)_2_D_3_, FGF-23, or PTH levels across tertiles of calciuria in the current series. Moreover, a former study by our group showed increased serum 1,25(OH)_2_D_3_ (within normality range) and monocyte expression of the vitamin D receptor (VDR) in SFs when compared to healthy subjects, irrespective of their values of urinary calcium [26]. On the other hand, in a previous immunohistochemical analysis of bone tissue from hypercalciuric SFs conducted by our group, Menon et al. [9] showed a significant positive correlation between sclerostin immunostaining in bone with serum 1,25(OH)_2_D_3_ in idiopathic hypercalciuric patients, which is in accordance with previous experimental data [27]. Therefore, it is possible that the tubular effects of sclerostin on the tubule are direct and not through any of the abovementioned mechanisms.

Regarding body composition, the current findings of a direct correlation of fat mass and inverse correlation of lean mass with serum sclerostin only among men agree well with previous studies [14,15], albeit this has been observed in women [28]. The negative association between sclerostin and muscle mass can be explained, at least in part, by an experimental study showing that older *Sost* KO mice exhibited the expected increases in bone mass but a significant reduction in whole-body fat mass and a strong trend toward increased lean body mass fraction [16]. 

Finally, the present data did not show differences in BMD according to tertiles of calciuria in both sexes. Despite the well-known association of low BMD with hypercalciuria, as suggested by most studies [6,10,29], it still remains a controversial matter as many investigators have reported low BMD among normocalciuric individuals as well [12], which is in agreement with the present findings of a lack of differences in BMD according to the levels of calciuria. In a very recent cross-sectional study of our group employing HRpQCT in SFs, Esper et al. [11] observed a reduced trabecular number (Tb.N) and volumetric BMD, indicating trabecular bone microarchitecture impairment, especially among women, as well as reduced bone strength parameters in men. Interestingly, they observed through a multivariate analysis an independent association of Tb.N with urinary calcium only at the distal radius, although BMI was a strong predictor of Tb.N at both radius and tibia. Such findings suggest that the mechanical loading to the tibia bones might have counteracted the calciuric effect at this site. Therefore, we considered the BMD/BMI ratio to be more adequate for comparisons with urinary calcium. However, although we observed a significant inverse association between the lumbar spine BMD/BMI ratio (*p* = 0.02, Table 3) with urinary calcium and a trend for the same association in total femur site (*p* = 0.06, Table 3) in univariate analysis, these two variables did not remain in the multivariate model. Interestingly, the use of a monoclonal antibody against sclerostin in healthy postmenopausal women has induced a dose-related reduction in mean urinary calcium levels [30]. 

Nevertheless, our study has some limitations. First, due to the cross-sectional nature of our study sample, it is not possible to prove causalities. This is a single-center-designed study, and thus the results could not be generalized to the overall population. Also, the present sample is relatively small. On the other hand, to the best of our knowledge, this is the first study to disclose an association of sclerostin and urinary calcium in stone formers.

In conclusion, in addition to hypertension and elevated salt intake, circulating sclerostin was shown to be a strong and independent determinant of urinary calcium among stone-forming patients. Moreover, sclerostin was correlated with body composition. These data suggest that in addition to the hormones traditionally thought to alter calcium reabsorption in the kidney, sclerostin may play a significant additional role, possibly intermediated by body composition, warranting further intervention studies in order to test potential medication strategies to reduce calciuria in this population. 

## Figures and Tables

**Table 1:** Clinical characteristics and nutritional data of SFs clustered into tertiles according to urinary calcium.

	Men			Women		
	T1 n = 19 (≤180 mg/24 h)	T2 n = 20 (180.1–283.0 mg/24 h)	T3 n = 19 (≥283.1 mg/24 h)	T1 n = 16 (≤150 mg/24 h)	T2 n = 17 (150.1–289.0 mg/24 h)	T3 n = 16 (≥289.1 mg/24 h)
Age, years	35.2 ± 7.9	38.3 ± 9.8	38.0 ± 10.3	31.6 ± 9.3	33.8 ± 8.3	37.4 ± 9.2
Duration of disease, years	9.0 (4.0–14.0)	9.0 (1.3–14.0)	7.0 (1.0–17.0)	9.0 (2.3–13.5)	5.5 (1.3–13.8)	3.5 (2.0–20.0)
BMI (kg/m^2^)	26.3 ± 4.8	26.6 ± 3.8	29.4 ± 4.3 ^ab^	24.6 ± 2.5	26.4 ± 5.1	27.8 ± 5.3 ^a^
Fat mass (%)	23.4 ± 7.3	22.6 ± 6.8	25.2 ± 5.4 ^c^	33.7 ± 6.4	33.8 ± 7.4	36.0 ± 4.8
Lean mass (%)	73.7 ± 6.7	74.6 ± 6.9	71.8 ± 5.0 ^ab^	63.7 ± 6.9	63.5 ± 7.3	61.4 ± 4.8
Hypertension, n (%)	0 (0)	3 (15.0) ^a^	5 (26.3) ^a^	1 (6.3)	1 (5.9)	6 (37.5) ^ab^
Metabolic syndrome, n (%)	2 (10.5)	5 (25.0) ^a^	8 (42.1) ^a^	1 (6.3)	2 (11.8)	6 (37.5) ^ab^
**Nutritional data**						
PNA (g/day)	1.0 ± 0.2	0.9 ± 0.4	1.1 ± 0.2	0.8 ± 0.5	1.0 ± 0.4	1.0 ± 0.4
NaCl (g/day)	12.9 ± 4.4	14.0 ± 5.6	15.5 ± 5.3	9.8 ± 4.2	10.5 ± 3.0	14.1 ± 4.5 ^ab^
**Energy-adjusted daily intake**						
Calcium (mg)	490.0 ± 165.6	569.3 ± 205.3	443.9 ± 201.2	638.6 ± 268.1	470.7 ± 145.0	469. ± 271.2
Phosphorus (mg)	984.2 ± 276.7	1021.7 ± 198.3	1026.8 ± 160.4	926.5 ± 200.2	1003.4 ± 189.6	1065.8 ± 222.7
Potassium (mEq)	52.8 ± 19.1	53.0 ± 9.4	53.8 ± 11.4	45.5 ± 9.4	46.9 ± 8.9	53.4 ± 12.3

^a^ vs. T1 *p* < 0.05; ^b^ vs. T2 *p* < 0.05; ^c^ vs. T2 *p* = 0.05. ANOVA with Bonferroni post hoc for normally distributed data and Kruskal–Wallis for non-normally distributed data. BMI, body mass index; PNA, protein equivalent of nitrogen appearance; NaCl, sodium chloride.

**Table 2:** Biochemistry and BMD data of SFs clustered into tertiles according to urinary calcium.

		Men		Women		
	T1 n = 19 (≤180.0 mg/24 h)	T2 n = 20 (180.1–283.0 mg/24 h)	T3 n = 19 (≥283.1 mg/24 h)	T1 n =16 (≤150 mg/24 h)	T2 n =17 (150.1–289.0 mg/24 h)	T3 n = 16 (≥289.1 mg/24 h)
**BMD parameters**						
Lumbar spine BMD (g/cm^2^)	0.99 ± 0.13	0.94 ± 0.08	1.04 ± 0.17	0.98 ± 0.03	0.94 ± 0.02	1.04 ± 0.04
Femoral neck BMD (g/cm^2^)	0.83 ± 0.16	0.82 ± 0.11	0.93 ± 0.18	0.83 ± 0.04	0.82 ± 0.03	0.93 ± 0.04
Total femur BMD (g/cm^2^)	0.97 ± 0.16	0.97 ± 0.12	1.06 ± 0.17	0.88 ± 0.10	0.88 ± 0.09	0.95 ± 0.14
**Urinary parameters**						
Calcium, mg/24 h	135.9 ± 29.6	236.9 ± 31.1 ^a^	363.4 ± 80.0 ^ab^	115.6 ± 26.8	217.6 ± 44.0 ^a^	341.6 ± 58.2 ^ab^
Oxalate, mg/24 h	22.2 ± 9.6	26.9 ± 8.4	28.6 ± 8.8	19.7 ± 7.0	20.0 ± 5.2	22.9 ± 7.5
Phosphate, mg/24 h	853.1 ± 268.6	963.8 ± 283.0	1204.8 ± 303.7 ^ab^	593.9 ± 203.0	707.7 ± 127.8	886.6 ± 228.9 ^ab^
Sodium, mEq/24 h	220.2 ± 74.8	237.2 ± 94.9	262.8 ± 90.0	166.8 ± 71.2	178.2 ± 50.3	239.3 ± 76.6 ^ab^
Potassium, mg/24 h	56.4 ± 22.4	65.2 ± 22.3	62.2 ± 25.6	44.7 ± 16.3	47.7 ± 12.7	51.7 ± 12.4
eGFR, ml/min/1.73 m^2^	97.3 ± 19.0	98.7 ± 15.5	96.3 ± 15.0	103.7 ± 13.3	102.4 ± 11.2	101.3 ± 10.9
**Serum parameters**						
Sclerostin (pmol/L)	21.4 (18.3–26.7)	28.8 (19.0–40.9) ^a^	30.9 (25.4–37.1) ^ab^	19.8 (11.0–23.9)	20.5 (17.5–23.0)	25.9 (17.5–29.4) ^c^
25 Vitamin D, ng/mL	24.0 (20.0–29.0)	23.5 (20.8–30.3)	25.5 (19.8–29.3)	27.5 (21.0–35.0)	25.0 (22.0–29.0)	26.0 (21.0–34.0)
1–25 Vitamin D, pg/mL	30.7 (17.3–39.5)	22.0 (16.6–50.8)	26.4 (18.0–34.6)	20.9 (17.8–27.2)	20.4 (15.1–33.0)	28.6 (21.1–60.5)
PTH. pg/mL	53.0 (40.0–67.0)	52.0 (37.5–64.0)	59.0 (42.3–76.5)	50.0 (45.0–60.0)	44.0 (41.0–65.5)	52.5 (35.5–65.0)
BAP, U/L	16.4 ± 5.8	16.5 ± 4.7	16.2 ± 4.1	13.0 ± 2.7	13.6 ± 4.7	13.3 ± 3.1
FGF-23, pg/mL	34.3 (31.5–42.1)	34.2 (26.6–47.9)	33.1 (26.4–39.8)	31.2 (24.8–39.3)	28.3 (24.7–36.3)	34.4 (19.7–44.4)
Klotho, pg/mL	684.0 (520.0–974.0)	730.0 (518.3–801.7)	714.0 (570.7–870.7)	615.4 (438.2–888.5)	829.5 (569.7–1221.3)	875.7 (508.2–1247.7)

^a^ vs. T1 *p* < 0.05; ^b^ vs. T2 *p* < 0.05 ^c^ vs. T1 *p* = 0.06. ANOVA with Bonferroni post hoc for normally distributed data and Kruskal–Wallis for non-normally distributed data. BMD, bone mineral density; eGFR, estimate glomerular filtration rate; PTH, parathyroid hormone; BAP, bone alkaline phosphatase; FGF-23, fibroblast growth factor 23.

**Table 3:** Potential determinants of urinary calcium.

	Total			
Potential Determinants	Univariate		Multivariate *	
	St. β	*p*	St. β	*p*
Age, years	0.26	<0.01	-	-
Sex, F	−0.11	0.25	-	-
Hypertension, yes	0.33	<0.01	0.30	<0.01
Fat mass, %	0.07	0.46	-	-
Lean mass, %	−0.08	0.41	-	-
NaCl intake, g/day	0.31	<0.01	0.31	0.02
PNA, g/kg/day	0.01	0.95	-	-
Calcium intake, mg/day	−0.19	0.11	-	-
Serum sclerostin, pmol/L	0.31	<0.01	0.26	0.01
Serum 1–25 vitamin D, pg/mL	0.02	0.87	-	-
Serum PTH, pg/mL	−0.05	0.62	-	-
Serum BAP, U/L	0.04	0.72	-	-
FGF-23, pg/mL	−0.08	0.43	-	-
Lumbar spine BMD/BMI ratio	−0.24	0.02	-	-
Femoral neck BMD/BMI ratio	−0.13	0.19	-	-
Total femur BMD/BMI ratio	−0.18	0.06	-	-

Linear regression analysis with serum sclerostin as dependent variable. * Run backwards, variables with *p* < 0.10 in univariate analysis included. Abbreviations: St. β, standardized beta; NaCl, sodium chloride, PNA, protein equivalent of total nitrogen appearance; PTH, parathyroid hormone; BAP, bone alkaline phosphatase; FGF-23, fibroblast growth factor 23.

## Data Availability

The data that support the findings of this study are available from the corresponding author, upon reasonable request.
